# Understanding HIV-positive patients' preferences for healthcare services: a protocol for a discrete choice experiment

**DOI:** 10.1136/bmjopen-2015-008549

**Published:** 2016-07-18

**Authors:** Elaney Youssef, Vanessa Cooper, Alec Miners, Carrie Llewellyn, Alex Pollard, Mylene Lagarde, Memory Sachikonye, Caroline Sabin, Claire Foreman, Nicky Perry, Eileen Nixon, Martin Fisher

**Affiliations:** 1 Department of HIV/GU Research, Brighton and Sussex University Hospital NHS Trust, Brighton, UK; 2 London School of Hygiene & Tropical Medicine, London, UK; 3 Department of Primary Care & Public Health, Brighton & Sussex Medical School, Brighton, UK; 4 UK Community Advisory Board, London, UK; 5 UCL Medical School, London, UK; 6 NHS England, London, UK

**Keywords:** PRIMARY CARE, GERIATRIC MEDICINE

## Abstract

**Introduction:**

While the care of HIV-positive patients, including the detection and management of comorbidities, has historically been provided in HIV specialist outpatient clinics, recent years have seen a greater involvement of non-HIV specialists and general practitioners (GPs). The aim of this study is to determine whether patients would prefer to see their GP or HIV physician given general symptoms, and to understand what aspects of care influence their preferences.

**Methods/analysis:**

We have developed and piloted a discrete choice experiment (DCE) to better understand patients' preferences for care of non-HIV-related acute symptoms. The design of the DCE was informed by our exploratory research, including the findings of a systematic literature review and a qualitative study. Additional questionnaire items have been included to measure demographics, service use and experience of non-HIV illnesses and quality of life (EQ5D). We plan to recruit 1000 patients from 14 HIV clinics across South East England. Data will be analysed using random-effects logistic regression and latent class analysis. ORs and 95% CIs will be used to estimate the relative importance of each of the attribute levels. Latent class analysis will identify whether particular groups of people value the service attribute levels differently.

**Ethics/dissemination:**

Ethical approval for this study was obtained from the Newcastle and North Tyneside Research Ethics Committee (reference number 14/NE/1193). The results will be disseminated at national and international conferences and peer-reviewed publications. A study report, written in plain English, will be made available to all participants. The Patient Advisory Group will develop a strategy for wider dissemination of the findings to patients and the public.

**Strengths and limitations of this study**

- This is the first study to use a discrete choice experiment (DCE) to investigate HIV-positive patients' preferences for service delivery, thereby involving service users in the design of future services in a way that is robust and easy to interpret.
- Recruitment will take place across 14 different sites including areas of high and low HIV prevalence and at sites with differing patient characteristics, thereby allowing comparison of preferences across different demographic groups. Recruiting sites include HIV clinics in London, Brighton and across the Kent, Surrey and Sussex network to ensure representation of patients who access different types of HIV clinics (eg, multicentre and single-centre clinics and smaller, rural clinics), and clinics with different links to general practice (eg, Brighton which has a locally enhanced service offering HIV-specific training to general practitioners (GPs)). The research benefits from a multidisciplinary collaboration of HIV clinicians (HIV doctor, nurse and pharmacist), health economists, health service researchers, statisticians, NHS commissioners, a GP and patient representatives.
- The attributes and levels have been chosen based on the findings of a systematic review and qualitative research to identify which aspects of care are most important to patients. To ensure the research can realistically inform the future design of services, we have selected attributes and levels which are modifiable and can be delivered within the context of the current Department of Health Policy and British HIV Association Guidelines.^1^ ^2^
- The design of the questionnaire requires participants to make a decision on what they might do, given a scenario, not what they have performed; a limitation of the methodology.

## Introduction

The number of people with HIV accessing services in the UK continues to rise.^3^ While mortality rates have dramatically improved as a result of increasingly effective antiretroviral treatment, they remain higher than in the general population.^4^ Increased survival, combined with later age at diagnosis, has resulted in a disproportionate growth in the proportion of older individuals accessing care.^5^

Complications due to opportunistic infections and malignancies associated with HIV are now rare in successfully treated individuals.^6^ ^7^ However, diseases not historically associated with HIV, but which are well recognised as complications of ageing, including cardiovascular disease, bone mineral density loss, cancer, cognitive decline, and hepatic and renal dysfunction, are increasingly identified. These comorbidities (defined as the presence of more than one distinct condition in an individual)^8^ appear to be occurring more frequently and at an earlier age in HIV-positive individuals than in HIV-negative populations.^9–15^ Older age and comorbidities are associated with polypharmacy and an increased risk of drug–drug interactions, which may lead to reduced efficacy of antiretroviral therapy.^16–18^

The care of HIV-positive individuals has historically been provided within specialist HIV clinics. Little is known about where HIV-positive individuals would prefer to access care for non-HIV-related acute symptoms. However, HIV specialists may not have the necessary experience to manage and treat these symptoms or comorbidities and a different model of care may be preferable from a clinical and patient perspective. Over recent years, alternative models of care have been suggested,^19–21^ such as combined clinics including one or more specialist in addition to the HIV clinician;^22^ HIV specialists adopting another specialist role (eg, with a special interest in renal or liver disease)^23^ and dedicated clinics for enhanced screening for comorbidities.^24^ However, these models have been developed in the absence of an evidence base and without consideration of patients' preferences. At a more strategic level, the recent NHS Five Year Forward Review emphasises the need for closer working relationships between primary and secondary care organisations.^25^ Thus, while it would appear that care arrangements for people living with HIV need to and are likely to radically alter over the next decade, the significant question of ‘how’ they should change, and whether changes would be welcomed from a patient perspective, remains.

To date, there has been very little research assessing patients' preferences for the delivery of healthcare among people with HIV. The systematic review suggested that valued aspects of care among HIV-positive individuals can be grouped into seven main themes: a good healthcare professional–patient relationship, HIV specialist knowledge, continuity of care, ease of access to services, access to high-quality information and support, effective coordination between HIV specialists and other healthcare professionals, and involvement in decisions about their treatment and care.^26^ There is currently no other study which has quantitatively captured patient preferences for healthcare in HIV-positive individuals in this way.

Involving patients in decisions about their care increases satisfaction with services, and improves attendance and adherence to treatment thereby improving health outcomes.^27^ Good management of chronic disease, including the management of comorbidities, is essential for the well-being of patients and reduces costs for the NHS.^25^ ^28^ ^29^ This study represents a first step towards developing a coherent, evidence-based model for the future management of HIV.

## Aims

The overall aim of the study is to determine whether patients would prefer to see a general practitioner (GP) or HIV physician given general symptoms, and to understand what aspects of care influence their preferences. Specific objectives are:

1. To determine the relative importance of different service attributes to people living with HIV.
2. To determine which measurable patient-related factors (including age, gender, ethnic group, sexual preference, quality of life and experience of other health conditions) influence preferences.

## Methods and analysis

A discrete choice experiment (DCE)^30^ will be conducted to better understand HIV-positive patients' preferences for their healthcare in relation to where they would choose to seek care given general symptoms. DCEs have long been used in health economics as a method for assessing patients' preferences regarding healthcare.^31^ This method is based on the premise that healthcare services can be described in terms of their attributes or characteristics and that an individual's evaluation, and therefore the choice of healthcare service, is based on these attributes. The key to assessing the relative preference for each attribute level is to generate a series of choices in a way in which respondents are required to ‘trade them off’ against each other. For example, a person could choose to see an HIV physician rather than a GP, but they may have to wait longer to see them. In this situation, the trade-off is between the type of healthcare provider seen (HIV physician or GP) and the waiting time.

### Choice of attributes and levels

The DCE uses a ‘labelled’ design, where the labels refer to choosing to go to the GP or an HIV clinic. The DCE attributes and associated levels were determined based on in-depth exploratory work, conducted as part of this NIHR funded research (Research for Patient Benefit). A systematic literature review was initially conducted to identify which aspects of healthcare are most important to people living with HIV. Subsequently, 12 focus groups consisting of a total of 74 patients were conducted with HIV-positive patients across South East England. Groups were quota sampled to ensure representation of younger and older individuals (<50 and ≥50 years), people from African and non-African communities, men who have sex with men, and heterosexual men and women. Participants were asked about their experiences of HIV and non-HIV healthcare services. Focus groups were audio-recorded and transcribed verbatim. Anonymous transcripts were analysed using a framework approach to establish the attributes and levels to be included in the DCE.

Following the exploratory phase, a comprehensive list of service attributes and levels for the DCE was produced. A process of selecting and refining these followed. Decisions on the final attributes and levels chosen from this list were made by the multidisciplinary Steering Group. Candidate attributes were prioritised if they could potentially be modified. For example, ‘trust’ emerged as being of particular importance to people with HIV; however, this was excluded from the DCE design on the basis that it is not readily amenable to intervention. However, as a way of trying to incorporate this into an attribute which could be modified, how many times the participant had seen the healthcare provider in the previous year was included as an attribute in the final questionnaire. The other attributes included in the final DCE were the healthcare professionals' expertise in managing general medical problems, HIV-specific expertise, ability to refer the patient on to other services, availability of appointments, how quickly appointments are made available and level of access to the patient information. The healthcare professionals' expertise in managing general medical problems was the only fixed attribute, which means that the answers for this particular attribute did not vary. This allowed the participant to understand that the GP was a generalist with general knowledge, whereas the HIV clinician was a specialist healthcare professional and did not therefore have the same level of generalist knowledge as a GP. The attributes and levels used in the final DCE are summarised in table 1.

**Table 1:** Attributes and levels used in the final DCE

Attribute	GP levels	HIV clinic—levels
The person you see is skilled at managing many general medical problems	▸ Yes	▸ No
The person you see has the ability to refer you on to another healthcare professional, if required	▸ Yes, to any specialist doctor	Yes, but only to your GP Yes, to any specialist doctor
How quickly you will be seen	The same day The next day In 7 days In 14 days	The same day The next day In 7 days In 14 days
An appointment out of usual working hours if you would like it	Not available Yes, 8:00 to 20:00 7 days a week Yes, Monday to Friday 17:00–20:00 Yes, Saturday 8:00—mid-day	▸ Not available
Whether you have seen the healthcare professional before	No never Yes, once in the last year Yes, twice in the last year Yes, more than twice in the last year	No never Yes, once in the last year Yes, twice in the last year Yes, more than twice in the last year
The type of person you see	A GP with specialist HIV training A GP without specialist HIV training	An HIV consultant doctor A doctor training to specialise in HIV An HIV specialist pharmacist An HIV specialist nurse
The level of information the person has access to	All your medical records, including your HIV details All your medical records, except your HIV details	All your medical records, including your HIV details Just your HIV medical records

DCE, discrete choice experiment; GP, general practitioner.

Each of the DCE questions are framed in the same way, so participants are presented with a hypothetical scenario where they are asked to imagine that they have woken up with a symptom such as a headache, fever, rash, diarrhoea or abdominal pain. Given this scenario, they are then asked to look at the attributes and levels of each clinic and place a tick under the clinic they would prefer to visit. These symptoms were chosen on the basis of an audit of 50 sets of patient notes looking at the most common reasons for walk-in or telephone triage HIV emergency appointment requests by patients experiencing a new symptom that could be easily managed by a non-HIV specialist. The results will therefore not only indicate the relative importance of service attribute, but rather will give an indication of how a GP service will have to look in order for HIV-positive patients to attend with a new, acute symptom.

**Figure 1:**
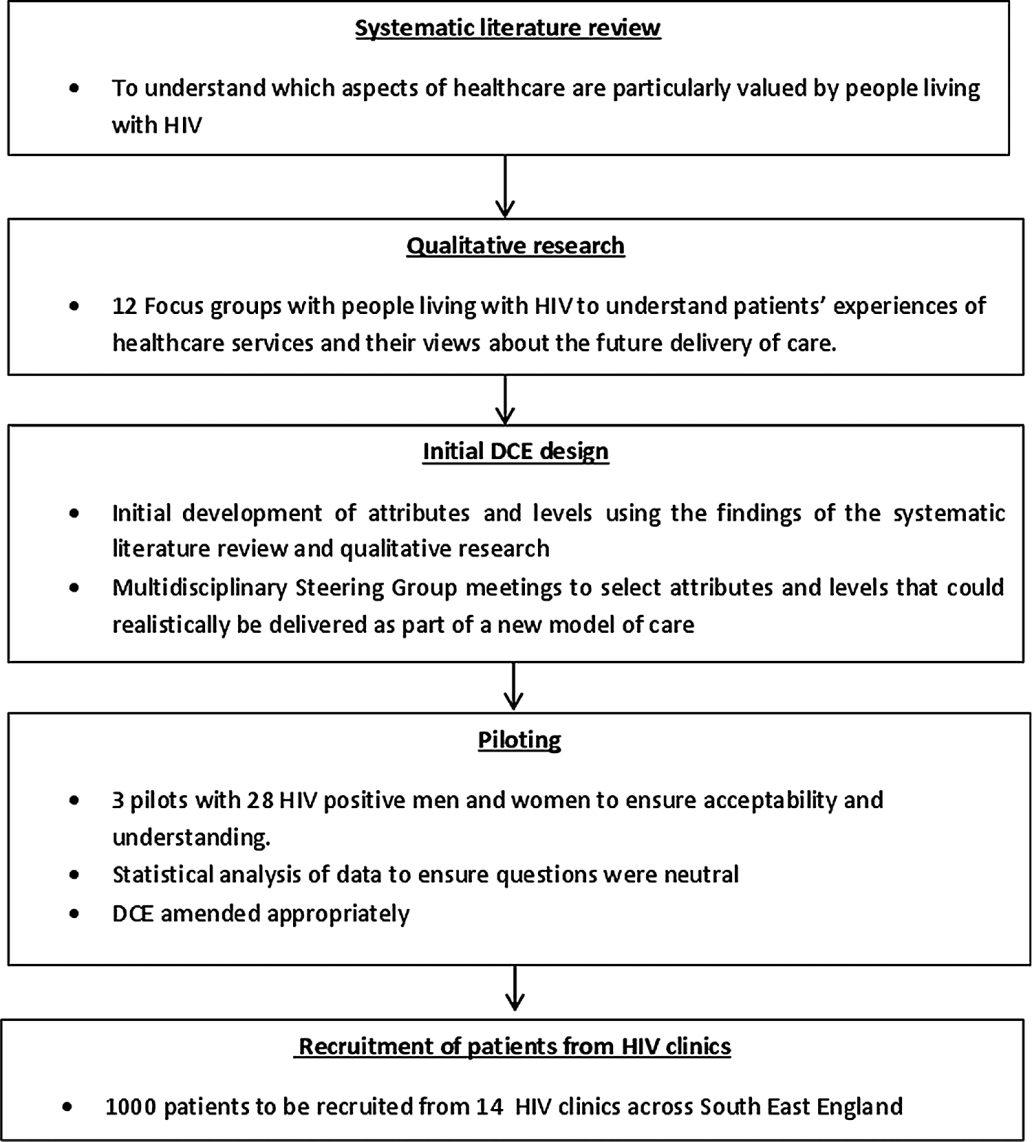
Development of the discrete choice experiment.

Early drafts of the DCE questionnaire were developed and reviewed by the multidisciplinary research team. The DCE was piloted three times with a total of 28 HIV-positive people recruited through community organisations, to ascertain patient acceptability and understanding. The questionnaires were revised following each pilot study. Data from the final pilot were analysed to look for dominance in each attribute and level to ensure that the questions were neutral and did not lead participants to choose one service over another. An overview of the DCE development process can be seen in figure 1. The initial DCE questionnaire was generated using an orthogonal approach with zero priors, and set to 24 choice tasks given the number of questions and 21 df in the design. The final design used a Bayesian D-efficient approach; the results of a pilot study consisting of 28 HIV-positive people were used to inform the priors.

The number of attributes and levels chosen resulted in a total number of 24 possible choices/questions. To limit the number of questions/time burden on participants, these questions have been split into 2 blocks of 12 and each participant will be asked to complete one block picked at random. Assigning attributes and levels to each block is performed by rotating each option to ensure a balance in each block. To reduce the impact of ‘learning effects’, there are two versions of each block; both containing the same questions but in reverse order.

**Figure 2:**
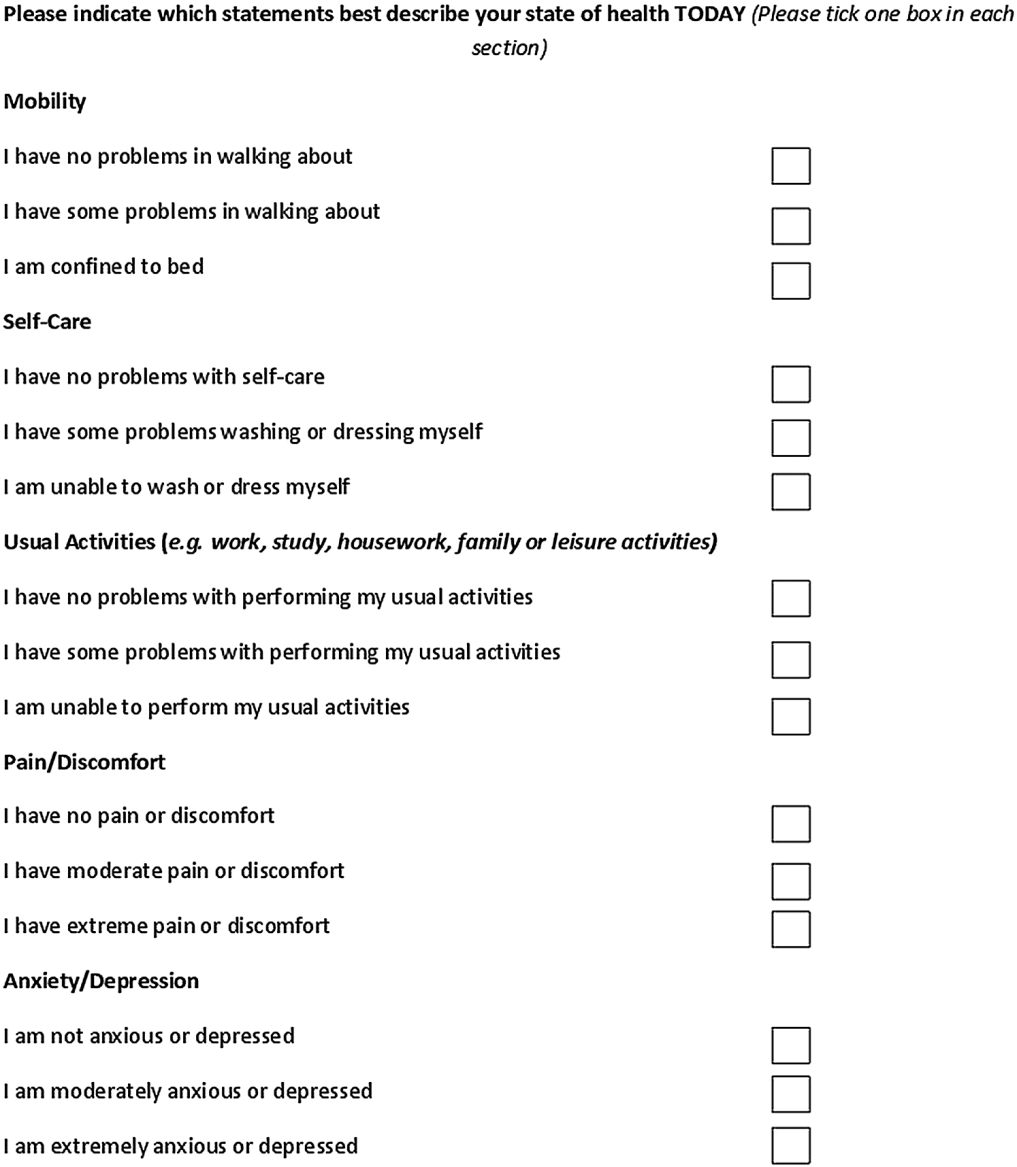
EQ5D-3L questions included in the final discrete choice experiment.

Demographic questions containing items on age, gender, sexuality, ethnicity, highest qualification and working status, GP service use, whether GP is aware of HIV status and sexuality, self-reported clinical information (including HIV treatment status, current and nadir CD4, and year diagnosed with HIV) and self-report of any current comorbidities have also been included to understand whether preferences differ depending on measurable patient characteristics. A further five questions from the EQ5D-3L health questionnaire^32^ have been included to assess the quality of life (figure 2).

## Sample size and power

Sample size calculations for DCE experiments cannot be calculated before the number of attributes and levels has been decided unless there is sufficient information. However, there is doubt about the relevance of sample size calculation in this methodology, because differences in the strength of preferences for attributes are not always expected. Since sufficient information is lacking, a power calculation was not performed. However, previous studies using DCE methodology typically include 300–400 participants.^33^ We have therefore set our recruitment target at 1000 participants; 500 completing ‘block 1’ questionnaires and 500 completing ‘block 2’ questionnaires which is beyond the target needed to power the study. This sample size will enable us to compare preferences for services between different demographic groups.

Clinic throughputs and rates of attendance indicate that more than 15 000 potentially eligible service users will attend participating sites over a 6-month period. Previous uptake of questionnaire-based research at these sites suggests that at least 50% of eligible patients approached will complete and return the questionnaire.

## Administration and analysis of the questionnaire

This study will be conducted in 14 HIV clinics across the Kent, Surrey and Sussex Clinical Research Network, and London. This will increase the generalisability of the findings, as recruitment will take place in areas of high and low HIV prevalence, rural and urban settings with different models of HIV clinics (eg, multicentre and single-centre clinics), in areas with and without enhanced GP services and in areas with varying patient demographics.

The questionnaire is anonymous and patients will therefore not be required to complete any information that could disclose their identity. All clinical information is obtained by self-report by the patient and medical notes will not be accessed. The location of questionnaire completion will be recorded on all questionnaires to assess differences in patient preference depending on where the patient attends for HIV care.

## Identification and recruitment of patients

Patients will be asked to confirm their eligibility for the study by confirming they:

- are aged 16 or over and
- have been diagnosed with HIV for at least 1 year.

Questionnaires and study materials are available in English and French. Patients attending HIV clinics at participating sites will be recruited in a variety of ways, as described below.

- Patients recruited at their HIV clinic will be given verbal study information by their clinical team, research nurse or study dedicated researcher. Those expressing an interest in taking part will be asked two screening questions in order to determine eligibility and (if applicable) will be given the study questionnaire to complete. Participants will be encouraged to complete the questionnaire in the clinic and once completed, to return it to a box in the clinic area to maintain anonymity. Patients who express a preference to complete the questionnaire online will be directed to it via a Quick Response code (a type of matrix barcode) or a web address, which will take them directly to the online version of the questionnaire. Patients identified by the clinical team who have previously provided written consent to receive information about research studies by email will be sent an email invitation by the research team. This will include study information and a link to the questionnaire.
- Posters will be put up in participating HIV clinics. These posters will include study information, screening questions and information about how to access the questionnaire. The online version of the questionnaire will ask the participant which clinic they attend for their HIV care from a drop-down list of participating clinics, and this will allow questionnaires completed online to also be traceable to a particular clinic.
- Study cards (business card size) will be available at each clinic's reception area. They will provide information about how to access the questionnaire online.

## Data analysis

Data will be entered directly by one of the study researchers into Microsoft Access, and 10% will be checked by a second researcher. A pragmatic decision was made that if the error rate exceeded 1%, then further verification by a second researcher would be undertaken. Data will be imported into SPSS V.20 and frequency distributions will be used to check for outliers. The data will be stored on NHS computers within the study team organisations and will be password protected.

The responses to the DCE questionnaires will be analysed using conditional logit and latent class models (LCM); the latter allows for serial correlations in responses and a formal assessment of preference heterogeneity. The LCM groups respondents into classes that have similar preferences and in so doing, identifies participant characteristics associated with particular group membership. All of the main effects parameters will be assumed to be alternative specific, meaning that respondents could value an attribute differently depending on whether it related to a GP appointment or HIV-clinic appointment. The data will be analysed using STATA V.13.1 and NLOGIT V.5.

Results of this study will inform HIV-positive patients' preferences for future care models. The analysis of preferences by demographic group will ensure that all patients' preferences are represented and will allow healthcare models to be tailored to meet the needs of these patient groups.

## Dissemination

There will be no formal written consent process, as consent will be implied by completion of the questionnaire. Patients will be assured that their answers are confidential. They will be asked to confirm their eligibility for the study by answering two screening questions. Participants will be provided with a brief description of the study and instructions for completing the questionnaire, including an example DCE question on the questionnaire itself. Contact details of the study researcher will be provided. The risks to patients of completing this anonymous questionnaire are minimal. However, should patients wish to discuss issues that arise during this research, they will be directed to speak with their usual HIV doctor or nurse who will ensure that appropriate support services are made available.

A study report, written in plain English, will be made available to all participants. Submission of an abstract to the British HIV Association conference and publication in a peer-reviewed HIV journal will follow. The Patient Advisory Group will develop a strategy for wider dissemination of the findings to patients and the public.
